# Individual-specific and subgroup level associations between stress and psychopathology in daily life: A temporal network investigation

**DOI:** 10.1192/j.eurpsy.2021.393

**Published:** 2021-08-13

**Authors:** R. Groen, C. Arizmendi, K. Gates, M. Schreuder, M. Wichers, C. Hartman, J. Wigman

**Affiliations:** 1 Department Of Psychiatry, Interdisciplinary Center Psychopathology And Emotion Regulation (icpe), University of Groningen, University Medical Center Groningen, Groningen, Netherlands; 2 Department Of Psychology And Neuroscience, University ofNorth Carolina at Chapel Hill, Chapel Hill, United States of America

**Keywords:** cross-diagnostic, Stress reactivity, person-specific analysis, temporal network analysis

## Abstract

**Introduction:**

Stress is a risk factor for developing psychopathology. Emerging evidence suggests that daily experiences of stress may also predict symptoms during the day. It is unclear to what extent the influence of stress on psychopathology during the day is the same across individuals (including across diagnostic boundaries), and which effects are individual-specific

**Objectives:**

This study aims to reveal how stress and symptoms are interrelated in a cross-diagnostic context by modeling individual level temporal networks, and examining subgroups with similar dynamics.

**Methods:**

Hundred twenty two young adults (43.4% women) with a wide range of psychopathology in terms of severity and type of problems completed a six-month daily diary study. We used a temporal network approach (i.e., group iterative multiple model estimation) to model how stress and ten specific symptoms (e.g., feeling down, paranoia, restlessness) were related across time at the individual-specific, subgroup, and group level.

**Results:**

After controlling for the lagged influence of stress on itself, stress level predicted the level of restlessness, worrying, nervousness, and feeling down during the same day for >70% of individuals. We observed three larger subgroups with each over 20 individuals, whose temporal networks showed different dynamic patterns involving specific symptoms. Effects of stress on other specific symptoms differed across individuals, and these were not subgroup-specific.

**Conclusions:**

This study showed important overlap between individuals in terms of impact of stress on psychopathology in daily life. Subtle differences between individuals were also observed. Possibly, such differences are relevant for examining individual-specific vulnerability for future psychopathology. This requires further investigation.

**Disclosure:**

No significant relationships.

